# The Usefulness of High-Frequency Ultrasound in Assessing Complications After Minimally Invasive Aesthetic Medicine Procedures, Using the Example of Assessing Blood Flow in the Dorsal Artery of the Nose

**DOI:** 10.3390/diagnostics16020271

**Published:** 2026-01-14

**Authors:** Robert Krzysztof Mlosek

**Affiliations:** Diagnostic Ultrasound Laboratory, Medical University of Warsaw, 03-242 Warsaw, Poland; mpage@wp.pl; Tel.: +48-601355236

**Keywords:** complications, dermal filler, high-frequency ultrasound, skin ultrasound, threads, ultrasonography, aesthetic medicine

## Abstract

In recent years, there has been rapid growth in aesthetic medicine and an increase in the number of minimally invasive procedures aimed at improving appearance. With the increasing number of procedures performed, the incidence of post-operative complications is also rising, and high-frequency ultrasound (HFUS) is increasingly being used to assess these complications. The article presents the case of a 52-year-old woman who reported for an HFUS examination several months after non-surgical nose correction with hyaluronic acid (HA) and implantation of polydioxanone (PDO) lifting threads. The patient experienced post-treatment complications in the form of erythema, oedema and pain, followed by blanching and bruising of the skin. Hyaluronidase and prednisone were used for treatment. Four months after the procedure, the patient returned for another HFUS examination because, despite the disappearance of most symptoms, uneven purple-blue discoloration of the skin on the nose and a subjective feeling of cold persisted. At the time of the HFUS examination, the discoloration was barely visible. The grey-scale HFUS examination revealed foci corresponding to HA deposits and PDO threads located in close proximity to the dorsal artery of the nose. A Doppler examination revealed blood flow disturbances in this artery, which may indicate compression by the threads and be the likely cause of the patient’s complaints. High-frequency ultrasound has proven to be a useful diagnostic method for assessing such complications. Due to its safety, non-invasiveness and high reliability, HFUS has the potential to become a common diagnostic tool in aesthetic medicine practice.

**Figure 1:**
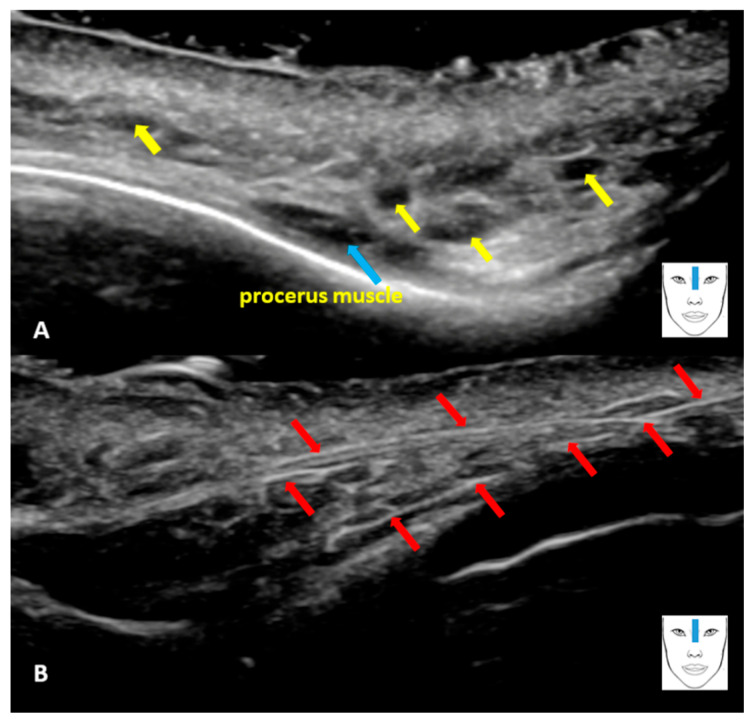
HFUS examination performed on a 52-year-old female patient 4 months after non-surgical nose correction, which consisted of the administration of hyaluronic acid and the implementation of PDO threads. Immediately after the correction, the patient developed symptoms indicating a complication. There was severe erythema, swelling, pain, followed by blanching, bruising and skin induration. The patient was injected with hyaluronidase twice. The procedure was performed only on the basis of palpation, ‘blindly’ without ultrasound control. In addition, the patient was given prednisone at a dose of 40 mg for the first 2 days, 20 mg for the next 2 days and 10 mg for the next 3 days. After treatment, most symptoms subsided; however, a reticular purple-blue discoloration of the patient’s skin remained, and she experienced a sensation of coldness in the nose, prompting her to undergo an HFUS examination. At the time of HFUS, these changes were barely visible, but the patient claims that they were more intense earlier. The HFUS examination was performed using a classic ultrasound scanner (Samsung RS85 Medison Co., Ltd., Republic of Korea) equipped with a linear ‘hockey stick’ transducer LA3-22AI with a frequency range of 3–22 MHz. For the examination, the “small parts” preset available on the ultrasound system was used and optimized to meet the examiner’s needs. The penetration depth was set to 1.5 cm. The examination was performed by a physician with many years of experience in this field. The examination was performed at the root of the nose; the location of the transducer is shown in the diagram above. The grey scale examination allowed for the imaging of focal lesions, which correspond to HA deposits (**A**). Blue arrow—procerus muscle. The deposits are visible as oval or ellipsoidal anechoic structures (yellow arrows) located in the subcutaneous tissue, well demarcated from the adjacent tissues [1,2]. The presence of HA deposits indicates ineffective administration of hyaluronidase, which is probably due to the fact that it was administered ‘blindly’ without ultrasound monitoring. The HFUS examination also revealed numerous linear, hyperechoic structures, whose image corresponds to the implanted PDO threads ((**B**), red arrows). The threads are located in the subcutaneous tissue, and HFUS allows their position relative to other structures to be traced.

**Figure 2:**
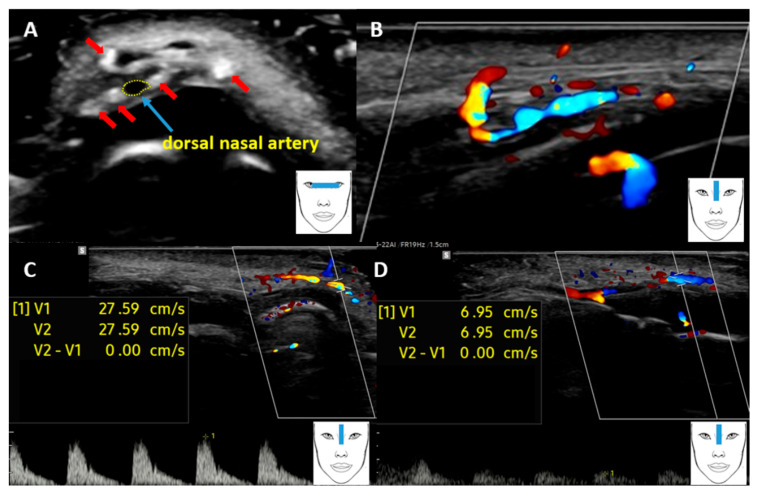
After placing the ultrasound transducer transversely to the root of the nose (Figure 1B), several strands were visualised in cross-section (red arrows), which are visible as hyperechoic oval-shaped structures. In addition, a blood vessel running between the strands was observed ((**A**), blue arrow), which was also imaged in cross-section. Based on anatomical knowledge, this vessel was identified as the dorsal artery of the nose. The flow spectrum obtained in the Doppler examination also confirmed that the imaged vessel was an artery (**C**). HFUS in greyscale showed that PDO threads are implemented right next to this artery. However, in the colour Doppler examination, this artery was visualised in a longitudinal section (**B**), where it is clearly visible that the artery passes between the implemented PDO threads, which lie very close to it. At the point where the artery was closest to the threads, the pulsed Doppler option was used to measure the flow velocity (V1, V2) through the artery, which was 27.59 cm/s (**C**). However, the flow velocity measurement (V1, V2) performed beyond the site of probable compression indicates that the flow is almost four times slower, at 6.95 cm/s (**D**). The result obtained indicates that in the area of increased flow there is pressure on the vessel, probably caused by a thread implanted too close, which in turn leads to inadequate blood supply to the tissues and may be the cause of the patient’s complaints of slight bruising of the nose and a feeling that the nose is cold. The images presented and the case described are probably the first reported case where threads implanted too close together contributed to impaired flow. However, there are reports in the literature confirming the relationship between extravascular pressure on arteries, mainly due to hyaluronic acid deposits, and skin ischaemia, skin discolouration and skin necrosis [3,4]. This case also points to the usefulness of HFUS in assessing complications after aesthetic medicine procedures. As this study has shown, Doppler options, which allow blood flow through the vessel to be monitored, have proven particularly useful [5,6]. The obtained HFUS findings may have significant clinical relevance, as they allow not only identification of the cause of vascular complications but also an informed selection of further therapeutic management. Precise localization of hyaluronic acid deposits and the course of PDO threads in relation to vascular structures enables decision-making regarding targeted administration of hyaluronidase or the need for thread removal. These procedures may be performed under real-time ultrasound guidance, which increases their effectiveness and safety while minimizing the risk of further vascular complications. HFUS may therefore play a key role not only in diagnosis but also in planning and monitoring the treatment of complications following aesthetic medicine procedures. As a safe, non-invasive, and reliable modality, HFUS has the potential to become widely adopted in aesthetic medicine practice.

## Data Availability

The original contributions presented in this study are included in the article. Further inquiries can be directed to the corresponding author.
